# Community Perceptions on Health Conditions Related to Indoor Air Pollution Among Adults Living in Urban Informal Settlements in Mwanza City, Tanzania

**DOI:** 10.24248/eahrj.v8i1.751

**Published:** 2024-03-28

**Authors:** Happyness Kunzi, Erica Sanga, Sospatro Ngallaba, George PrayGod

**Affiliations:** a Clinical Research Programme, Mwanza Research Centre, National Institute for Medical Research, Mwanza, Mwanza, Tanzania; b Department of Epidemiology, Behavioural Sciences and Biostatistics, School of Public Health, Catholic University of Health and Allied Sciences, Mwanza, Tanzania

## Abstract

**Introduction:**

Indoor Air Pollution (IAP) from biomass fuel is one of the major health threats globally. There is limited data on community awareness and perceptions of health conditions associated with IAP in urban informal settlements in sub-Saharan Africa. We explored community perceptions of IAP-associated health conditions, risk behaviors, and potential interventions to reduce IAP in urban informal settlements.

**Methods:**

We used purposive sampling to recruit participants from households located in Mwanza urban informal settlements. We conducted 16 In-depth Interviews (IDIs), two Focused Group Discussions (FGDs), and four Key Informant Interviews (KIIs). Obtained data were then transcribed, translated, coded and analyzed thematically with Dedoose qualitative data analysis software.

**Results:**

Majority of participants were unaware of the health conditions associated with IAP. Participants perceived biomass fuel from charcoal as a safe fuel compared to other known fuels (firewood and gas). Indoor biomass fuel use for cooking and use of rubber and plastic materials for fire lighting were the commonly practices and risk behaviors for IAP. Moreover, poverty is what guides the choice of fuel use for cooking.

**Conclusion:**

Participants awareness health effects of biomass fuel was low, strategies to reduce poverty and health promotion on the health effects of IAP are urgently needed in the Mwanza urban informal settlements.

## INTRODUCTION

Indoor air pollution (IAP) from biomass fuel is a major health threat as it is associated with various health risks^1^. Globally, about 3.8 million people a year die prematurely due to diseases associated with IAP^2^. IAP is generated when biomass fuels are burnt in poorly ventilated houses,^3^ resulting in smoke accumulation that contains Particulate Matters (PM) and harmful fumes like gaseous pollutants which contain carbon monoxide and formaldehyde.^4^ Exposure to these fumes and PM is associated with higher risk of both communicable and non-communicable diseases (NCDs).^5–7^ About 90% of rural households in low-and middle-income countries (LMICs) rely on biomass fuel for cooking and therefore, IAP is more common in these settings.^8,9^

Inability to afford alternative energy could be one of the major reasons for using biomass fuel for cooking,^10^ although poor awareness and perceptions on IAP risks could be contributing to perpetual use of biomass fuel in these settings,^8^ but data are limited.

This study aimed at understanding the perceptions on the health conditions associated with the use of biomass fuel and exploring community practices, risk behaviors and potential interventions to reduce IAP in informal settlements of Mwanza city.

## METHODS

### Study setting

This study was conducted in Nyamagana and Ilemela districts, which constitute Mwanza city. Mwanza city is the capital and the headquarters of Mwanza Region, located on the southern shores of Lake Victoria. The city covers 1,325 km^2^; and it has a total population of 706,453 of which 75% live in urban informal settlements. Mwanza city is highly populated with a population density of 150 people per km^2^. Most people in Mwanza city are self-employed in petty jobs, and some work in services sectors.^11^

### Study Population

The study population included adults (age ≥18years) from households living in urban informal settlements in Nyamagana and Ilemela district who were using biomass fuel for cooking purposes.

### Study Design

This was a qualitative, cross-sectional study conducted in urban informal settlements in Mwanza city. Data were collected, from July to August 2021. This study was nested in the large cohort study entitled Chronic Infections, Co-morbidities and Diabetes in Africa (CICADA) (NCT03106480). CICADA is an observational study investigating the burden of, and risk factors for diabetes among 1947 adults with and without HIV in Mwanza, a region which is a major consumer of solid fuel in Tanzania with 40% of its population living in informal settlements.^10^

### Selection of Study Participants

Purposive sampling method was used to recruit participants for In-Depth Interviews (IDIs), Focus Group Discussions (FGDs), and Key Informant Interviews (KIIs). We used the CICADA cohort as a platform for identifying study participants from urban informal settlements found in Mwanza city to participate in this study. The CICADA cohort collected demographic data of participants including residential addresses of those known to be from urban informal settlements like Igogo, Mabatini, Mkuyuni, Mjimwema and Bugarika areas. CICADA project also collected data on the type of fuel used for cooking in the households and obtained permission from study participants to use these data for future research. Therefore, we used CICADA database to identify potential participants for this study. For IDIs, we recruited 16 participants who reported using biomass fuel from the CICADA database residing in any of the identified informal settlements based on the two districts of Mwanza City. IDIs were conducted within participants' households, once at the household the interviewer asked the family member to appoint a senior member whom they believed could provide intelligible information on health regardless of gender since health awareness cuts across both males and females. Nine participants from each district (Ilemela and Nyamagana) residing in informal settlements who were reported to be using biomass fuel were selected to participate in FGDs. Eighteen (18) participants who reported using biomass fuel and residing among urban informal settlements were purposely selected from the CICADA cohort to participate in two FGDs. The selection of participants considered diversity in terms of age, gender, and marital status as well as settlements diversity to increase confidence in the data collected.^12^ In addition, four participants for KII were purposely selected from the two study districts in Mwanza city (Ilemela and Nyamagana) to get an insight of what is known and conducted in the community in relation to indoor air pollution and health outcomes. KIIs included one environmental management officer from Nyamagana municipal council who is responsible for overseeing issues related to air pollution. Likewise, we interviewed two community leaders (village chairpersons) from each district. Community leaders are selected by citizens; and are considered as community representatives. In addition, we interviewed one medical officer in charge from Buzuruga Health Centre, a health centre serving majority of informal settlement dwellers in Ilemela district.

### Inclusion and Exclusion Criteria

Participants aged >18 years old, living in Mwanza informal settlement and using biomass fuel for cooking were eligible for this study. People residing in urban formal settlements were excluded from this study.

### Data Collection Methods

Face to face interviews using interview guides with open-ended questions were used for data collection. All interviews were conducted in Kiswahili (Tanzanian national language); the interviews were audio-recorded after being granted permission by the participants. The IDIs and KIIs were conducted in a private room. For confidentiality, participants were given numbers for identification and were asked for their permission to use anonymous quotes. Data were collected by well-trained and experienced researchers to ensure good quality of data and equal chance of expressions from participants.^13^

The IDIs collected data on perceptions related to health conditions from IAP due to biomass fuel use as well as practices and risk behaviors that lead to IAP while FGDs collected information on social-cultural believes, norms, and values that could lead to IAP. Data from FGDs sought to understand the general community views on the IAP. KII collected data on informant views and opinions about IAP and the currently available strategies as well as the potential interventions for reducing IAP.

### Quality Assurance

To ensure credibility, confirmability and dependability of the study,^14^ data collection tools were constructed based on the study objectives and pre-tested prior to the study. Information was triangulated through IDIs, FGDs and KIIs. Data were collected by well-trained and supervised researchers for data dependability. In addition, transcripts were translated by competent individuals in both Swahili and English language and checked by the first author. To ensure quality of qualitative research, the study was tied with the standards for reporting qualitative data.^15^

### Data Management and Analysis

Audio tape recorder, field notes, and interview transcripts were kept in a locked cabinet. Recordings were transcribed verbatim in Kiswahili language before translation in English. Data were then coded inductively by two coders (HK and ES) to ensure data validity, reliability and trustworthiness of research conclusion.^16,17^ Thematic-content-analysis was undertaken with the use of Dedoose qualitative data analysis software.^18^ The interpretation of results was done by two authors and cross-checked by the last author considering triangulation of the information received and comparing the results with findings from literature review of other similar studies.

### Ethical Consideration

The study received ethical approval from the Joint CUHAS/BMC Research Ethics and Review Board (CREC). All potential participants received full disclosure of the study and written consent was sought from all potential participants before enrolment.

## RESULTS

### Results Overview

We conducted interviews with 38 participants from communities (16 IDIs 18 FGDs and 4 KIIs). Characteristics of those from communities are included in Table 1. Men were 55.3% and majority of participants were between 41-61 years of age. Majority were petty traders and attend to primary school, only a few attended secondary school without necessarily completing it. Most of households had 6-10 family members, only 7% of household used gas and charcoal for cooking and the remaining 93% of household depended on polluting fuel as a major source of cooking fuel. Majority of participants were cooking indoors (Table 1). Four major themes were identified in this study, these included: (1) Perceptions of heath conditions related to IAP from biomass fuel for cooking; (2) Community practices and reasons for using biomass fuel for cooking and cooking indoors; (3) Other practices leading to IAP and (4) Potential interventions for reducing IAP.

**TABLE 1:** Characteristics of Participants Involved in the Study (N=38)

Participants Characteristics	Number (%)
Sex	
Male	21 (55.3)
Female	17 (44.7)
Age groups	
20–40	7 (18.4)
41–60	25 (65.8)
≥ 61	6 (15.8)
Education level	
Not attended school	1 (2.6)
Attended primary school	28 (73.7)
Attended secondary school	6 (15.8)
Attended to college and university	3 (7.9)
Family size	
<5	11 (28.9)
6–10	27 (71.1)
Type of cooking fuel	
Charcoal only	20 (52.7)
Charcoal and gas	7 (18.4)
Charcoal and firewood	11 (28.9)
Cooking place	
Indoors	32 (84.2)
Outdoors (i.e. Kitchen)	6 (15.8)
Occupation	
Not working	4 (10.5)
Petty trader	23 (60.5)
Employed	8 (21.1)
Peasant	3 (7.9)

### Perceptions of Health Conditions Related to Biomass Fuel for Cooking

Majority of the participants (more than half1) were not aware of any links between IAP from biomass fuel and other various health conditions. Participants did not see the possibilities of having negative health conditions due to biomass fuel because they have never experienced any of the heath risks even after many years of biomass fuel use.

*“I have used firewood since I was a child …I have been using charcoal fuel in my household for many years, I have never experienced any negative health conditions and no one in my family has experienced negative effects…I have never seen any of my relatives feeling or having any of the mentioned health conditions*”. [FGD-Male]

*“I don't think biomass is such a threat to our health…biomass fuel is our life and we have been surviving on it…we cook and get some income from charcoal…all people in this community use biomass fuel and no one has ever complained about it”.* [IDI – Female]

In addition, few participants perceived the use of charcoal fuel being safe for cooking as it has no smoke hence no negative health conditions. The participant's felt the use of firewood has more risks to health conditions compared to charcoal.

*“Firewood fuel is the most harmful compared to charcoal as someone must sit near the cooking place so he/she inhales more smoke while with charcoal you don't need to sit near cooking stove during cooking”.* [IDI-Female]

On the other hand, four participants were able to mention at least one or two health conditions. The health conditions that were frequently mentioned by the few include chest tightness and chest pain, flue, red eyes and coughing.

*“I have been using charcoal for a very long time; there was a time when I had difficulty in breathing and I had headache for a long-time …A few days ago, headache and chest pain become so severe that I could not handle them…to my surprise the doctors told me to stop using charcoal fuel as it is the major cause for my illness”.* [IDI-Female]

*“Long exposure to smoke from biomass fuel leads to eyes irritation…eye scratching due to irritation causes eye redness… my grandmother was suspected to be a witch because she had red eyes which was the effect of biomass smoke”* [Male-FGD]

### Community Practices and Reasons of Using Biomass Fuel for Indoor Cooking

Participants reported some of the reasons for choosing to use biomass fuel over other sources of energy for cooking. Poverty was reported as a major reason for the use of biomass fuel in the study settings because it is cheaper and more easily accessible in urban areas.

*“It is cheaper and affordable even when someone has Tshs 1,000 or Ths 500 while with gas you need to have Tshs 18,000 to refill the gas… Charcoal is being sold at retail price; so it is affordable even with a small amount of money”.* [IDI-Male]

*“Charcoal fuel is being sold everywhere in our community…I think you have seen it while coming here…my two neighbours sell charcoal outside their house…with this, charcoal is easier to obtained in this community”.* [IDI-Female]

Majority of participants were against the use of alternative fuel like gas and electricity as they are expensive, not user friendly and gas is associated with house explosion as well as destroying aluminum pots.

*“Gas and electricity are less used in our community …We are scared of the gas and electricity explosion as well as cost for purchasing it…My house exploded a few years ago and since then I discourage the use of gas in my house*”. [FGD-Male]

*“Cooking using gas fuel is associated with damaging pots… at least biomass fuel use gives us a life relief in terms of purchasing new pots regularly”.* [IDI-Female]

Others felt biomass fuel suits big family size, easy to apply especially for the family with children and it can cook variety of food especially long-time cooking foods like beans and meat.

*“I like cooking with charcoal because it is easier for children to use, they can understand when to add more charcoal and still have time to play unlike gas and which requires someone to stay around when cooking”.* [FGD-Female]

*“I have a big family size…we cook using big pots which cannot fit on gas stove, only charcoal or firewood can accommodate our cooking pots”.* [IDI-Male]

Cooking indoors is a common community practice reported to increase the risk for IAP. Reasons for indoor cooking were, absence of space for outdoor cooking, outdoor kitchen in rented houses are limited as well as avoiding wind that leads to high charcoal consumption and security reasons.

*“We‘re many tenants in the same house or compound you cannot be free when cooking because there is no outdoor kitchen … it is hard to trust people since we have just met in a renting compound… so we decide to cook indoors”.* [FGD-Female]

Again, poverty was mentioned as a reason for indoor cooking in the urban informal settlements. Because of low economic status, people cannot afford renting or building big houses that has separate kitchen.

*“We have no choice for the place for cooking instead of cooking indoors… I cannot afford building a separate kitchen outside because there is no space and I don't have that kind of money… my only space is where the house is standing as you can see we don't have even a space for hanging washed clothes*”. [IDI-Male]

One landlord mentioned about tenants who purposely decide on destroying rented houses for reason of cooking indoors.

*“Tenants have no pain with the house floor… tenants do not consider if it is rainy or dry season, they choose to cook indoors as they have nothing to lose because they don't own the house or room and consider destroying the renting house as value for their money*”. [FGD-Male]

### Other Practices Leading to Indoor Air Pollution

The study found that some people were using plastics bottles for cooking when charcoal and firewood where not available; this was reported as one of the risk behaviours for IAP. Smoked fish sellers were reported using plastic bottles and artificial hair which give more smoke which could affect people nearby and fish end users.

*“People prepare smoked fish using plastic bottles and artificial hair as fuel as they can be found every where and they give a nice colour for the smoked fish…this is due to poverty and scarcity of firewood and charcoal…Plastic bottles have a lot of smoke but they cook fast”.* [FGD-Female].

It was also reported that materials used for igniting charcoal were among the risk practices that influence IAP; including using plastic bags, old slippers, old mattresses, torn clothes, and used masks. When used, these materials produce more and heavy smoke when they come into contact with fire. These igniting materials are often used in the informal settlements because they are easily accessible and it is considered to be a way for waste management

*“Lighting charcoal or firewood using kerosene is the best practice but due to poverty we cannot afford buying kerosene for this purpose…we normally use torn clothes, old slippers, mattresses and plastic bags to light our charcoal stove…These materials have more smoke but they are very effective”*. [FGD-Female]

### Recommended Strategies and Interventions to Reduce Indoor Air Pollution

Most participants thought that improvement of social economic status in urban informal settlements will greatly help in the reduction of IAP. People suggested that social support programmes that are being provided by the government reach all those in need. The poverty reduction strategy would enable majority of people in urban informal settlement to purchase gas and electric stoves as well as building and renting houses with separate kitchens.

*“The government should help people living in these poor communities to have a better economic status by expanding the Tanzania social support fund (TASAF) to reach more people so that we can also be able to purchase gas and electricity for cooking…We are not happy with the use of biomass fuel but due to poverty we cannot run from it”*. [FGD Male]

Participant suggested health education to raise awareness of biomass fuel among users on the potential risks associated with biomass fuel for cooking. Media broadcasting and brochures should be provided to people to remind them of the negative effects of biomass fuel for cooking. On the other hand, participants proposed education related to proper use of the modern fuel for cooking (gas and electricity) to reduce gas and electricity explosion risks. Some people are refraining from the use of these clean forms of fuels for fear of accidents.

*“Health education should be provided to biomass users on proper use of gas and electricity fuel as everything has negative impact if not handled well…We need to be educated on how to handle and how to cook using gas and electricity to reduce fear and avoid explosions”.* [FGD 1-Male]

Participants expressed the need for involvement of local leaders in enhancing the government proposed strategies for IAP reduction because they are closer to people in the society.

*“Local leaders are closer to the community and they know how to deal with their people…I suggest all the interventions initiated by the government for IAP reduction, should be directed to local leaders for implementation”.* [KII-Male]

## DISCUSSION

This study sought to understand the community perceptions on health conditions related to IAP and explored community practices and risk behaviors for IAP as well as potential interventions for IAP reduction. Majority of participants were unaware that exposure to biomass smoke could lead to various health conditions. Participants could not directly connect the health risks with exposure to biomass smoke during cooking. These findings are similar to those in studies conducted in other countries on IAP;^19,20^ and this is due to lack of knowledge on air pollution health risks.^21^ Most of our study participants (73.7%) had only attained primary education and might not have attended classes on health effects of environmental pollution. In addition, mass media platforms like radio and television are the commonest reliable source for spreading IAP health awareness,^22^ but majority of our study participants were petty traders and thus due to poverty, they may have not been able to buy radio and television sets to access health information. Although we did not collect updated data on asset ownership, in CICADA study, possession of radio and television were lower in those in the lowest tertile of social economic status (PrayGod G, personal communication). This suggests that, other forms of health communication like use of public meetings in places where petty traders meet such as local markets should be considered in planning education programmes on effects of IAP in poor urban settings.

Poverty seemed to be the major reason for biomass fuel use as well as indoor cooking; with high price of gas and electric fuel, majority of the people living in the urban informal settlements cannot afford these fuels. In addition, poor people cannot afford to rent complete houses or build bigger houses with separate kitchens in the urban informal settlements. These findings are similar to the qualitative study done in Nairobi among slum residents.^23^ Poverty in urban informal settlements is associated with inadequate house ventilation and use of unclean solid fuel for cooking.^24^ Thus increased IAP can be attributed to poverty in urban informal settlements.^25^ This suggests that, poverty reduction interventions should be included in IAP reduction strategies. Furthermore, this indicates that, biomass fuel use is inevitable in the urban informal settings due to poverty, hence campaigns against IAP should include strategies to improve charcoal and firewood stoves with low pollution level.

We also found that some people in urban informal settlements were reluctant to change from biomass fuel to clean forms of fuel especially gas fuel because they thought that electric and gas fuels are associated with fire accidents. This corroborates findings of similar to studies conducted in the city of Dar es Salaam in Tanzania and in north western part of Ghana.^26,27^ This perception could be attributed to lack of knowledge on IAP effects; in some people lack of knowledge on proper use of clear energy like electricity and gas may be the reason behind perpetual use of biomass fuel for cooking.

Cooking and lighting charcoal stoves using plastic and rubber materials like plastic bottles, old slippers, and plastic bags were found to be the risk behaviours that lead to IAP. These findings are similar to the review done in Nairobi slum residents-Kenya and in Kigali-Rwanda.^23,28^ Plastics materials are found everywhere for free and using plastics for igniting charcoal and firewood stove is sometime considered as a form of waste management.^29^ Smoke from plastics and rubber materials contains approximately 50%, benzene, 20% toluene, 25% xylene, and 5-6% ethylbenzene and they contain high amount of carbon monoxide, as well as particulate matter 2.5 micrometers missions.^30^ These are associated with higher risk of developing cancers, heart attacks, neurological, kidney and liver diseases.^31,32^ Studies to investigate the burden of use of plastics and rubber as fuel and link to health outcomes are urgently needed to help advocate for the need to rollout interventions to reduce the burden of IAP in Tanzania.

### Strengths and limitations

This study used different data collection methods which allowed validation and triangulation of information and therefore increased validity of the results. However, the results are limited to verifications, because participants had more control on the data collected and we could not verify the results objectively against the scenarios stated by the respondents

## CONCLUSION AND RECOMMENDATIONS

The study showed minimal awareness on health conditions related to IAP among biomass users, indoor cooking and poverty were the major contributors for IAP. Improvement in the economic status and education promotion on both health risks and proper use of gas and electricity stove could help in controlling the burden of IAP.
